# Feasibility of cardiac MR thermometry at 0.55 T

**DOI:** 10.3389/fcvm.2023.1233065

**Published:** 2023-10-03

**Authors:** Ronald Mooiweer, Charlotte Rogers, Rohini Vidya Shankar, Reza Razavi, Radhouene Neji, Sébastien Roujol

**Affiliations:** ^1^ School of Biomedical Engineering and Imaging Sciences, Faculty of Life Sciences and Medicine, King’s College London, London, United Kingdom; ^2^ MR Research Collaborations, Siemens Healthcare Limited, Camberley, United Kingdom

**Keywords:** cardiac, MR thermometry, low field MRI, interventional, MR guidance

## Abstract

Radiofrequency catheter ablation is an established treatment strategy for ventricular tachycardia, but remains associated with a low success rate. MR guidance of ventricular tachycardia shows promises to improve the success rate of these procedures, especially due to its potential to provide real-time information on lesion formation using cardiac MR thermometry. Modern low field MRI scanners (<1 T) are of major interest for MR-guided ablations as the potential benefits include lower costs, increased patient access and device compatibility through reduced device-induced imaging artefacts and safety constraints. However, the feasibility of cardiac MR thermometry at low field remains unknown. In this study, we demonstrate the feasibility of cardiac MR thermometry at 0.55 T and characterized its *in vivo* stability (i.e., precision) using state-of-the-art techniques based on the proton resonance frequency shift method. Nine healthy volunteers were scanned using a cardiac MR thermometry protocol based on single-shot EPI imaging (3 slices in the left ventricle, 150 dynamics, TE = 41 ms). The reconstruction pipeline included image registration to align all the images, multi-baseline approach (look-up-table length = 30) to correct for respiration-induced phase variations, and temporal filtering to reduce noise in temperature maps. The stability of thermometry was defined as the pixel-wise standard deviation of temperature changes over time. Cardiac MR thermometry was successfully acquired in all subjects and the stability averaged across all subjects was 1.8 ± 1.0°C. Without multi-baseline correction, the overall stability was 2.8 ± 1.6°C. In conclusion, cardiac MR thermometry is feasible at 0.55 T and further studies on MR-guided catheter ablations at low field are warranted.

## 1. Introduction

Cardiac catheter ablation is a well-established minimally invasive procedure to treat heart rhythm disorders, such as ventricular tachycardia (VT) (1). This technique uses radiofrequency energy to induce localized heating and scarring of specific myocardial tissue areas to correct problematic electrical pathways responsible for arrhythmias. VT ablation procedures are typically performed using electro-anatomic mapping and fluoroscopic guidance, but currently suffer from a high rate (up to 50%) of VT recurrence (2, 3). Potential causes for this high recurrence rate include inadequate ablation results, which can occur since the ablation efficacy is conventionally only monitored indirectly: for example through applied power, ablation duration, catheter tip temperature, applied force, and impedance, which are of limited predictive value to assess the extent of the permanent lesion (4, 5). Furthermore, the ionizing radiation in fluoroscopy can be harmful to both patients and staff.

MR-guided VT ablation is a promising alternative approach. MRI provides excellent soft tissue visualization as well as enabling assessment of the VT substrate and ablation lesion (6–8). Real-time ablation lesion imaging can be performed using MR thermometry (9), which could provide real-time feedback to the operator, potentially allowing instantaneous evaluation of the desired ablation position and depth by monitoring the accumulated lethal thermal dose (7, 10–13). Cardiac MR thermometry has been previously demonstrated at 1.5 T and 3 T using the proton resonance frequency shift (PRFS) (14) method combined with ECG-triggered single shot EPI (7, 12, 13, 15–17).

In the recent years, low field MRI (B_0_ < 1 T), which has a lower associated cost, has received renewed attention (18–20). Low field MRI is especially interesting for interventional MRI as more devices can be introduced safely with reduced heating sensitivity through RF energy deposition, whilst generating fewer susceptibility artefacts in the images. Furthermore, patient access is expected to be improved due to a generally larger bore size. Although MR thermometry has previously been shown at low field strengths in several organs (21, 22), its feasibility in the heart remains unknown.

The PRFS method relies on the temperature-induced offset in effective magnetic field strength which manifests as a localized linear phase signal variation when using a gradient echo sequence (14). Temperature maps can be obtained by subtracting the phase image at any time point during ablation from a baseline phase image acquired prior to hyperthermia. In CMR thermometry, an additional correction has to be made for breathing-induced magnetic susceptibility changes that lead to periodically changing phase maps and errors in temperature maps (15, 23–26). An effective correction approach is the multi-baseline method (23, 25). Here, multiple dynamic images are acquired prior to hyperthermia over several breathing cycles and are stored in a look-up-table. During hyperthermia, each incoming dynamic thermometry image is matched to one of the look-up-table acquired at the most similar breathing position, resulting in a phase difference and corresponding temperature map with minimal respiration-induced errors. The correction of respiration-induced phase changes for cardiac MR thermometry hasn't been described before at low field, where susceptibility effects are expected to be reduced compared to higher field strengths.

In this paper, the feasibility of cardiac PRFS thermometry was investigated *in vivo* on a commercially available 0.55 T wide bore MRI scanner. Healthy volunteers were scanned without heating source. The requirement for correction of respiration-induced susceptibility changes was studied by calculating temperature maps with and without multi-baseline correction. The stability of MR thermometry time-series was assessed by their standard deviation over time.

## 2. Methods

To assess the feasibility of cardiac MR thermometry at low field, 9 healthy volunteers (5 male, 32 ± 5 years old) were scanned. The study was approved by the Institutional Research Ethics Committee (HR-18/19-8700) with written informed consent obtained from all subjects. All imaging was performed on a 0.55 T MAGNETOM Free.Max MRI scanner (Siemens Healthcare, Erlangen, Germany), with an 80 cm bore and maximum gradient amplitude and slew rate of 25 mT/m and 40 T/m/s respectively. Coil arrays on the chest (anterior) and within the bed (posterior) were used for MR signal reception, and sequences were cardiac triggered using an external ECG device (Expression MRI Patient Monitoring System, Invivo, Orlando FL, USA). All images were obtained under free breathing conditions, the subjects were instructed to breathe steadily without sharp in- or exhalations.

**Figure 1:**
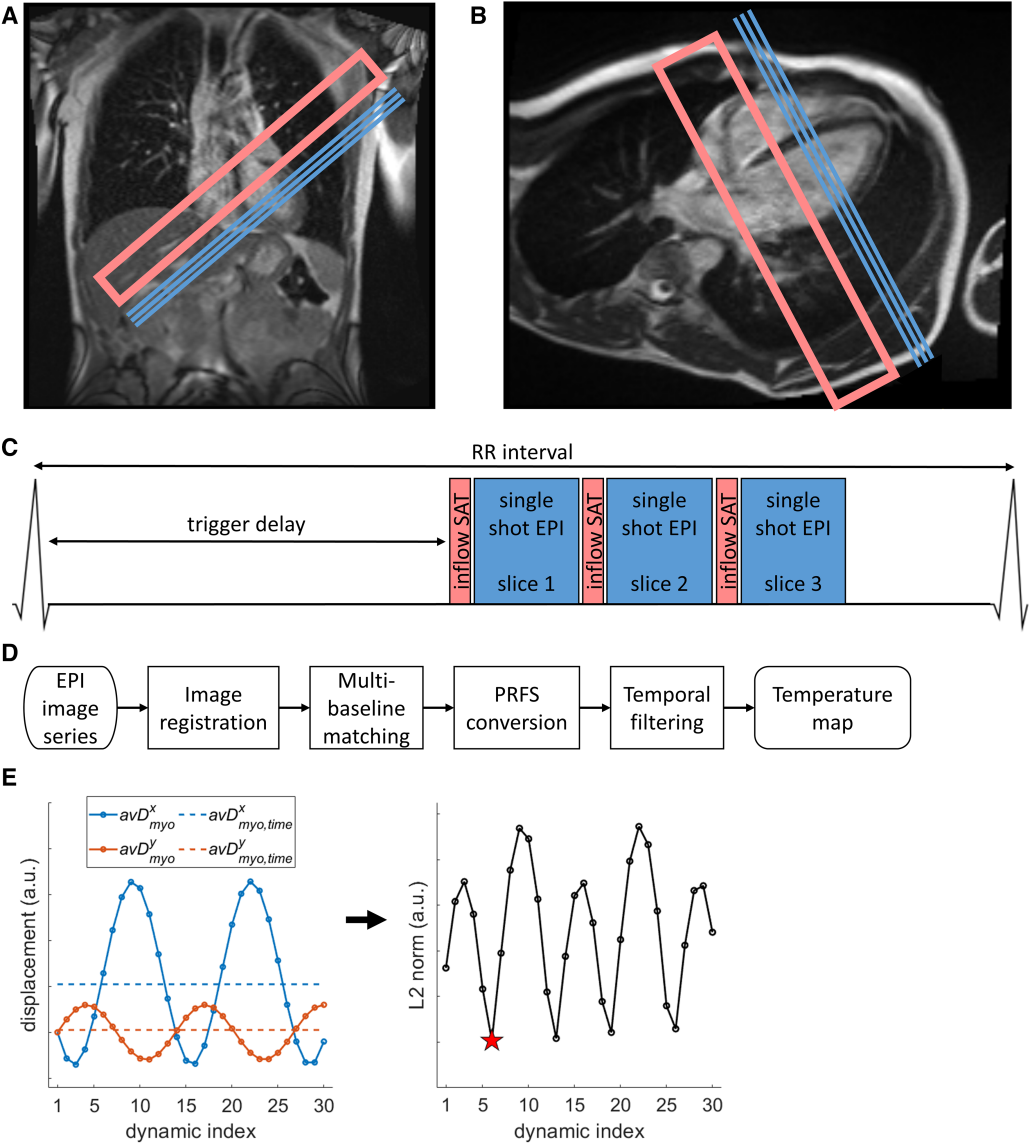
Illustration of cardiac MR thermometry methods. (**A,B)** Location of image slices (blue lines marking slice center lines) and inflow saturation (pink open rectangles) on coronal (**A**) and four-chamber (**B**) localizer images. (**C**) Pulse sequence representation of the cardiac-triggered imaging protocol. (**D**) Processing steps starting from scanner-generated EPI images to the final stability of thermometry value. (**E**) Illustration of mid-respiration detection steps. **Left**: average displacement over a myocardial mask (*avD^x/y^_myo_*, circles connected by lines, x/y coordinates in blue/orange) of the first 30 dynamics to the first dynamic, and the time-average over these 30 dynamics (*avD^x,y^_myo,time_*, dashed lines, x/y coordinates in blue/orange). **Right**: the dynamic index with minimal L2 norm between ***avD_myo_*** and ***avD_myo,time_*** was chosen as best representing the middle of the respiration cycle (red star).

A dynamic series (*N* = 150) of single shot EPI images was acquired for cardiac MR thermometry using the PRFS method (9), which is sufficiently long to cover the typical duration of an RF ablation (1). The sequence parameters were: FOV = 350 × 263 mm^2^, resolution = 2.2 × 2.2 mm^2^, slice thickness = 6 mm, GRAPPA acceleration factor = 2, bandwidth = 1,078 Hz/px, flip angle = 75°, TE = 41 ms, echo train duration = 66.5 ms. The flip angle was approximately the Ernst Angle (27) for the T_1_ of myocardium at 0.55 T and an assumed heart rate (repetition time) of 60 beats per minute. Inflow saturation using a saturation slab was applied for blood signal suppression, which has been shown to reduce partial volume effect and facilitate image registration during the reconstruction process (16). Slice coverage included 3 contiguous slices of the left ventricular myocardium, in short axis orientation. Imaging geometry and a sequence diagram are shown in Figures 1A–C. Note that a longer TE than prior higher field strength studies was selected to take advantage of the longer T_2_* of myocardium at 0.55 T [47 ± 4 ms vs. 30–37 ms at 1.5 T (18)], given that the precision of PRFS thermometry is maximized at TE = T_2_* (9).

Temperature-change maps were calculated offline using a modified version of a pipeline that has been shown to be executable in real-time (26) and is schematically represented in Figure 1D. As part of this pipeline, non-rigid image registration (28, 29) was applied to correct for respiration-induced in-plane motion and deformation, aligning the time-series in space to facilitate pixel-wise phase-change analysis. Then, multi-baseline matching (23, 25) was applied to correct for phase changes arising from respiration-induced changes of the local magnetic susceptibility (look-up-table length of 30 dynamics). Phase wraps were corrected for in the temporal domain and the remaining phase differences were converted to temperature changes using the PRFS method (9, 14). Temporal filtering of temperature maps using a window-based low-pass FIR filter (window: Kaiser, order: 11, cutoff frequency: 52 mHz at 6 dB attenuation) was applied to reduce noise, as previously described (26, 30).

As a brief expansion to the pipeline for more robust image registration, an image acquired near the middle of the breathing cycle (i.e., mid-way between end-inspiration and end-expiration) was identified and used as target/reference image for registration of the entire series (Figure 1E). To achieve this, the images within the multi-baseline set (first 30 dynamics) were initially registered to the first image of the series, resulting in displacement fields (***D***) for each dynamic image. The spatial average of the 2D displacement was then calculated for each dynamic image (***avD_myo_***). The time-averaged mean 2D displacement was also calculated as ***avD_myo,time_***. The dynamic image index representing the middle of the breathing cycle was approximated as the dynamic image for which the L2-norm of the vector difference between ***avD_myo_*** and ***avD_myo,time_*** was minimized. This image was then used as registration target for the entire thermometry image series.

The stability of MR thermometry, defined as the standard deviation of temperature change over time, was calculated for all voxels in the myocardium. Masks of the myocardium were manually drawn on each slice using images resulting from averaging the magnitude images across all dynamics. The average stability of MR thermometry over the myocardium of all slices is reported for each subject. This thermometry reconstruction pipeline was repeated without multi-baseline correction of respiration-induced phase changes to investigate its requirement at 0.55 T. To test if the thermometry stability with and without multi-baseline correction was significantly different, the Wilcoxon signed rank test was applied on the mean stability values of all subjects. *P*-values < 0.05 were considered significant.

## 3. Results

**Figure 2:**
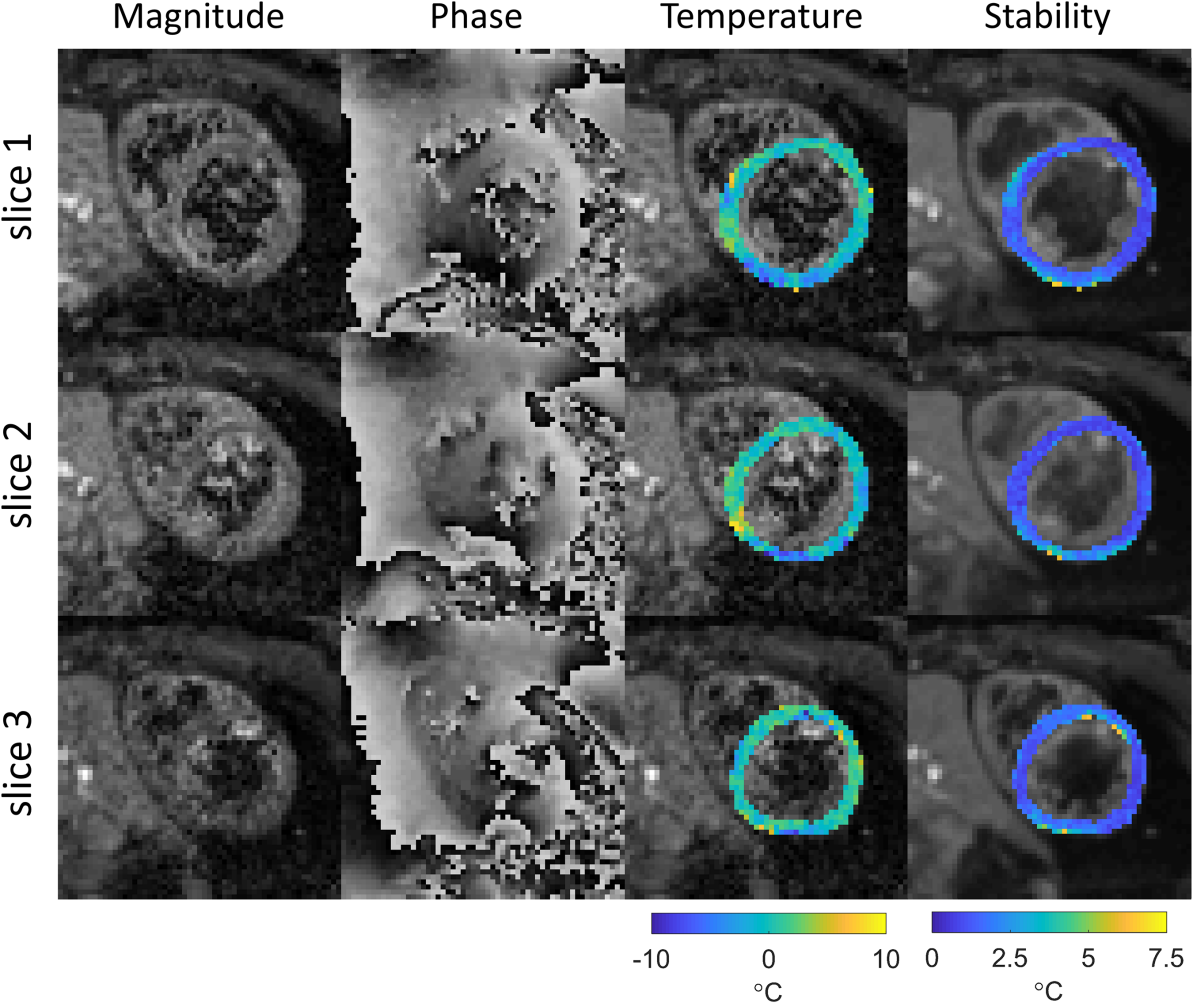
Example of thermometry results in subject 4. Magnitude and phase images (after image registration) and temperature change maps (after filtering) for the last dynamic image are shown for all slices, as well as the derived stability maps. The temperature maps are shown in color overlaid on grayscale magnitude images of the same dynamic acquisition, the stability maps are shown in color overlaid on grayscale images of the magnitude images averaged across all dynamics.

Cardiac MR thermometry imaging was successfully performed in all subjects. Example images are shown in Figure 2, including typical magnitude and phase images. Substantial blood signal reduction was achieved successfully throughout the left ventricular blood pool of all slices. Temperature and MR thermometry stability maps were found homogenous throughout all slices. In this subject, the stability of MR thermometry was 1.6 ± 0.9°C.

**Figure 3:**
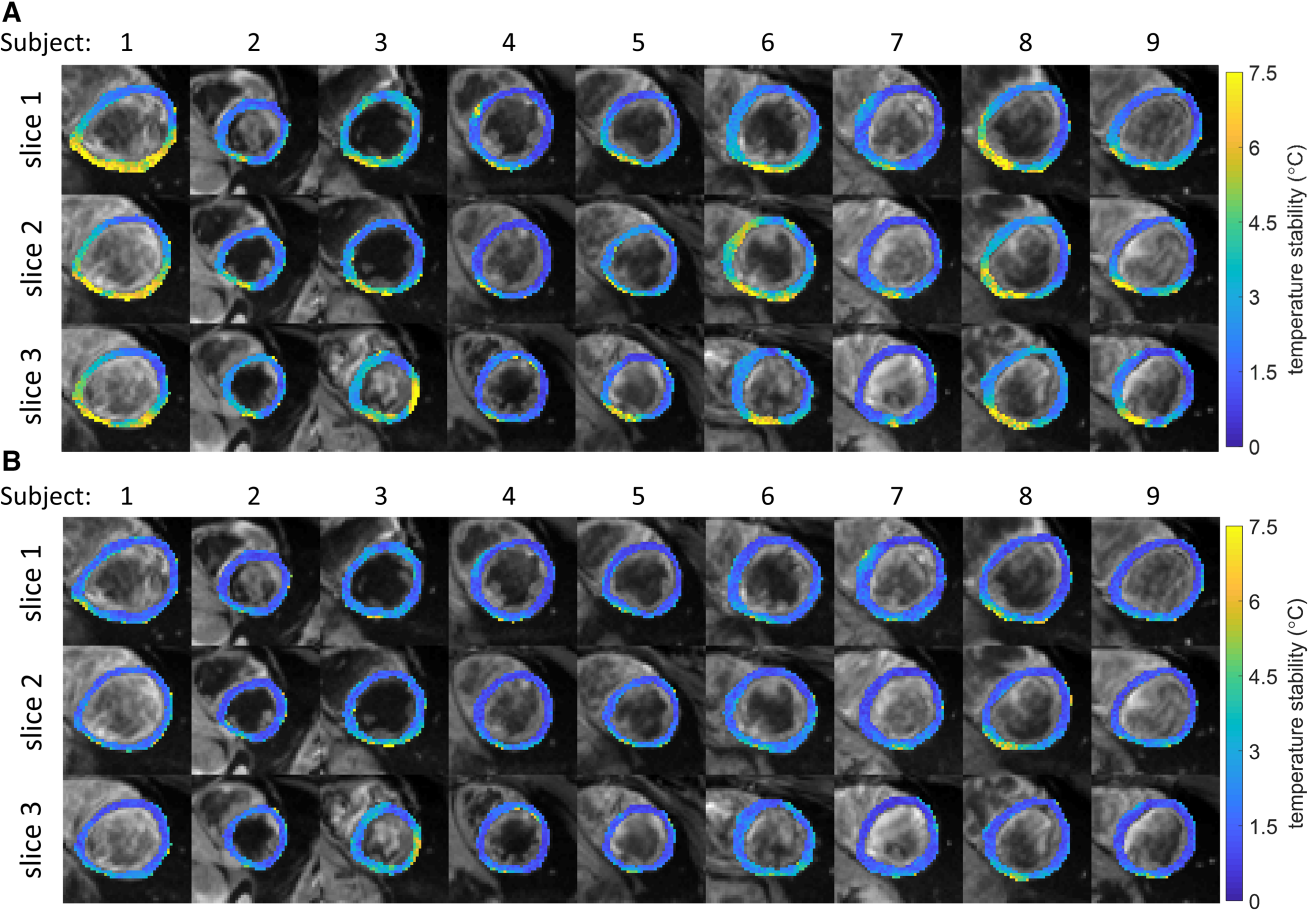
(**A,B**) Stability of temperature measurements for all subjects and slices (color). Black and white background images are the average magnitude images over all dynamics. (**A**) No multi-baseline correction was applied during temperature calculation, variations within and between subjects can be seen. (**B**) Multi-baseline correction was applied during temperature calculation, reduced variability can be seen compared to **A**. While most myocardial pixels display a stability of <3°C, localized regions of higher instability can be seen.

Maps of the stability of thermometry are shown in Figures 3A,B for all slices and subjects. Without multi-baseline correction (Figure 3A), larger inter and intra subject variations can be observed compared to the stability maps that were reconstructed with multi-baseline correction included in the thermometry reconstruction pipeline (Figure 3B). Using this full thermometry reconstruction pipeline (Figure 3B), stability of MR thermometry was consistent between all slices and subjects with homogeneous maps seen across most of the myocardium. In several subjects and slices a slight reduction in temperature stability was however observed near the inferolateral segment, at the lung-liver interface. Over all subjects, 0.17 ± 0.24% of voxels had a stability >7°C, with no voxels exceeding this limit in 5 out of 9 subjects.

**Figure 4:**
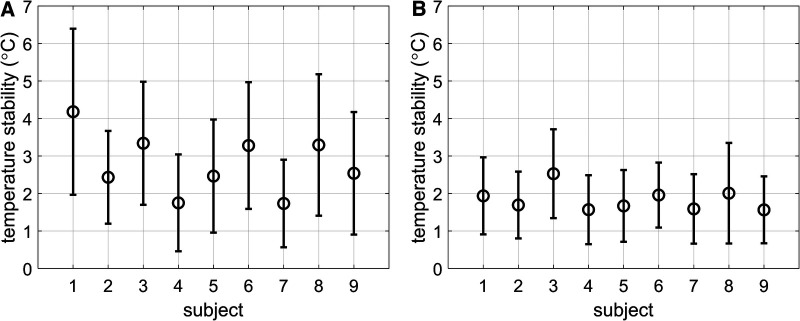
(**A,B**) Stability of temperature measurements reported as mean ± SD of the stability of all myocardial voxels per subject. (**A**) No multi-baseline correction was applied during temperature calculation, thereby allowing respiration-induced phase-variations to be considered as temperature changes. (**B**) Multi-baseline correction was applied during temperature calculation.

The stability of thermometry in the myocardium for all subjects is shown in Figures 4A,B, for thermometry reconstructions without (Figure 4A) and with (Figure 4B) multi-baseline correction. When multi-baseline correction is omitted from the reconstruction pipeline (Figure 4A), larger means and standard deviations can be seen for each subject, in line with the observations in Figure 3. Using the full thermometry reconstruction pipeline (Figure 4B), 7 out of 9 subjects displayed a mean stability below 2°C. Averaged over all subjects, the stability was 1.8 ± 1.0°C using the full thermometry reconstruction, compared to 2.8 ± 1.6°C when multi-baseline correction was omitted from the thermometry reconstruction. This difference was statistically significant (*p* < 0.004).

## 4. Discussion

*In vivo* cardiac MR thermometry was shown to be feasible at a commercially available 0.55 T low field MR scanner using the PRFS method and standard acquisition and reconstruction techniques used at higher field. Multi-baseline correction of respiration-induced phase changes was shown to be beneficial at this field strength.

Compared to a reported temperature stability at 1.5 T of 1.5 ± 0.4°C (13), the overall stability of 1.8 ± 1.0°C at 0.55 T was similar, although with a slightly larger variation. This similarity is encouraging for monitoring of ablation efficacy and warrants further studies into MR-guided ablations at this field strength. Other preliminarily reports of PRFS thermometry at low field in the brain and prostate were also favorable (31) and a similar trade-off between reduced SNR and improved choice of scan parameters has recently been reported for magnetic-particle-based MR thermometry (32). The lower intrinsic SNR and PRFS sensitivity (which is proportional to magnetic field strength) of low field MRI was expected to reduce the thermal stability compared to higher field strengths. The comparable result might be attributed to the longer TE which is made possible by the longer tissue T_2_*, and increased signal due to more T_1_ regrowth because of shorter T_1_ values (18, 19). However, this comparison between stabilities obtained at different field strengths is limited as it is not a direct comparison by changing field strength alone. Other differences between our study and the literature are in scanner hardware and software, the different thermometry reconstruction pipelines used, and inter-subject variabilities.

Signal loss and the corresponding reduced thermometry stability near the lung-liver interface is a known issue in cardiac imaging and thermometry in particular where a single-shot EPI sequence is used (12, 13, 15–17). Ablation for VT can be needed in any location in the LV, and despite the low field strength, the precision of thermometry was reduced near the lung-liver interface compared to the rest of the myocardium. Still, at 1.5 T, 3.7 ± 0.9% of the total myocardial voxels were considered too much degraded (stability >7°C) (13), whereas in this study at 0.55 T a smaller percentage of voxels met this criterion (0.17 ± 0.24%).

Blood signal reduction using inflow saturation was successful in most cases, although the signal was not fully suppressed. Alternative blood signal suppression strategies such as the double inversion recovery technique (33) have been found successful in this context (16, 34), although limiting the acquisition to a single slice or requiring simultaneous multi-slice acceleration techniques for multi-slice protocols.

Subject-specific characteristics such as heart rate and body mass index could also affect thermometry stability values. Although not reported specifically in the literature, one could expect a higher heart rate leading to reduced stability through the lower signal available due to shorter time available for T_1_-recovery. Similarly, a higher body mass index would generally be expected to lead to a reduced thermometry stability as noise levels are expected to increase due to an increased distance between receive coils and the heart. Assessing these relationships would require a greater sample size than presented in this feasibility study.

Registration of the images was performed to allow phase difference calculation of voxels representing the same tissue samples. However, no prospective through-plane motion correction (i.e., slice tracking) was applied. Slice tracking could be applied using diaphragmatic navigators (35) or active microcoils present near the catheter tip (36). Therefore, integration of prospective slice tracking will be the focus of future work, which is expected to further improve the performance of the sequence.

Retrospective correction of breathing-induced phase changes is considered crucial for cardiac MR thermometry (7, 12, 13, 15–17) and was evaluated here using the multi-baseline correction technique. Thermometry reconstructions without this correction degraded the overall stability from 1.8 ± 1.0°C to 2.8 ± 1.6°C. Even though susceptibility effects are generally expected to be reduced at low field, corrections of respiration-induced phase changes are still beneficial.

The low field scanner did not include native ECG triggering, and an external system was used. Scanner-implemented ECG triggering could be more robust in practice as it could reduce signal delay and could offer more advanced filtering of MR-sequence-induced interference on the ECG signal as well as arrhythmia detection. Alternatively, cardiac triggering based on the active tracking microcoils in ablation catheters has been shown to be feasible (37).

This study has several limitations. Primarily, the presented measurements of intrinsic feasibility of cardiac MR thermometry were conducted in the absence of an RF ablation. During ablation, the resulting tissue property changes are expected to have limited influence on the measurement of temperature change as the PRFS effect is largely independent of tissue type in all water-based tissues (9). This was confirmed by prior studies at 1.5 T which demonstrated excellent agreement between ablation lesions estimated from PRF thermometry and ground truth (7, 12, 13). Another factor not included in this study is the presence of catheters near the myocardium that can cause susceptibility artefacts. These are more pronounced at the longer echo times used in this study, but this effect is also mitigated by the lower static magnetic field strength. Therefore, the feasibility and characterization of cardiac MR thermometry in the presence of a catheter and during ablation remain to be investigated. Additionally, fat saturation was not employed in this study. Finally, in this study healthy volunteers were scanned and further evaluation in a patient population, including patients with VT or implanted devices are now warranted.

## 5. Conclusion

Cardiac MR thermometry is feasible on a commercially available 0.55 T scanner. Temperature stability of 1.8 ± 1.0°C was achieved in this study which is promising for the guidance of cardiac ablations at this field strength.

## Data Availability

The raw data supporting the conclusions of this article will be made available by the authors, without undue reservation.
